# The importance of rare species: a trait-based assessment of rare species contributions to functional diversity and possible ecosystem function in tall-grass prairies

**DOI:** 10.1002/ece3.915

**Published:** 2013-12-12

**Authors:** Meha Jain, Dan FB Flynn, Case M Prager, Georgia M Hart, Caroline M DeVan, Farshid S Ahrestani, Matthew I Palmer, Daniel E Bunker, Johannes MH Knops, Claire F Jouseau, Shahid Naeem

**Affiliations:** 1 Department of Ecology, Evolution and Environmental Biology, Columbia UniversityNew York, New York, 10027; 2 Northwest Institute for Plateau Biology, Chinese Academy of SciencesXining, 810008, China; 3 Department of Botany, University of HawaiiHonolulu, Hawaii, 96822; 4 Department of Biological Sciences, New Jersey Institute of TechnologyNewark, New Jersey, 07102; 5 Department of Biology, The Pennsylvania State UniversityUniversity Park, Pennsylvania, 16802; 6 School of Biological Sciences, University of NebraskaLincoln, Nebraska, 68588; 7 Muséum National d'Histoire Naturelle, Ecologie et Gestion de la BiodiversitéParis, 75231,, France

**Keywords:** Biodiversity–ecosystem functioning, community assembly, functional diversity, grassland, species loss, traits

## Abstract

The majority of species in ecosystems are rare, but the ecosystem consequences of losing rare species are poorly known. To understand how rare species may influence ecosystem functioning, this study quantifies the contribution of species based on their relative level of rarity to community functional diversity using a trait-based approach. Given that rarity can be defined in several different ways, we use four different definitions of rarity: abundance (mean and maximum), geographic range, and habitat specificity. We find that rarer species contribute to functional diversity when rarity is defined by maximum abundance, geographic range, and habitat specificity. However, rarer species are functionally redundant when rarity is defined by mean abundance. Furthermore, when using abundance-weighted analyses, we find that rare species typically contribute significantly less to functional diversity than common species due to their low abundances. These results suggest that rare species have the potential to play an important role in ecosystem functioning, either by offering novel contributions to functional diversity or via functional redundancy depending on how rare species are defined. Yet, these contributions are likely to be greatest if the abundance of rare species increases due to environmental change. We argue that given the paucity of data on rare species, understanding the contribution of rare species to community functional diversity is an important first step to understanding the potential role of rare species in ecosystem functioning.

## Introduction

The majority of species in ecosystems are rare, but the ecosystem consequences of losing rare species are poorly known (Lyons et al. 2005; Bracken and Low 2012). Biodiversity loss, defined here as local extinction, can influence ecosystem functioning if those species lost possess traits that directly or indirectly influence ecosystem function (Cardinale et al. 2006; Duffy 2009; Lewis 2009). Rare species may be at greater risk of extinction because of low abundances, small geographic ranges, and greater susceptibility to environmental and demographic stochasticity (MacArthur and Wilson 1967; Pimm et al. 1988, 1995; Hubbell 1997; Smith and Knapp 2003; Wilsey and Polley 2004). Yet experimental tests of the influence of biodiversity on ecosystem functioning (BEF) have primarily focused on common species (Lyons et al. 2005). Though it is true that common species may disproportionately affect ecosystem function given their greater abundances (Smith and Knapp 2003; Gaston 2010), rare species should not be ignored as some species may possess unique traits or have complementary effects with other rare species that influence ecosystem function despite their low abundances (Lyons and Schwartz 2001; Smith and Knapp 2003; Hooper et al. 2005; Mouillot et al. 2013). Rare species may also play an important role in the long-term stability of ecosystem functioning if they become more abundant due to environmental change (MacDougall et al. 2013). Such turnover is the basis of biological insurance (Lyons et al. 2005), ecosystem reliability (Yachi and Loreau 1999), and ecosystem stability (Griffin et al. 2009).

While several studies have attempted to understand the influence of nonrandom species loss on ecosystem functioning (Lyons and Schwartz 2001; Zavaleta and Hulvey 2004; Hillebrand et al. 2008; Isbell et al. 2008), these studies did not specifically focus on the loss of rare species and they also did not consider the importance of rare species over time. This may be because experimentally examining the role of rare species is relatively intractable. Rare species are generally poorly or completely unknown, are difficult to cultivate, are often protected making experimental manipulations difficult, and may have to be studied for prohibitively long periods to assess their role after temporal turnover. An alternative to long-term, experimental manipulations is to measure the contribution of rare species to ecosystem functioning by quantifying how rare species contribute to community functional diversity via community trait space (Cornwell et al. 2006; Mouillot et al. 2013); species with redundant trait values will contribute little to community trait space, whereas species with unique trait values will contribute significantly to community trait space. This metric is based on the idea that the influence of a species on ecosystem functioning is associated with the functional traits it exhibits (Lavorel and Garnier 2002; Garnier et al. 2004). Trait diversity is an important predictor of ecosystem function given that studies have found a positive relationship between functional diversity and ecosystem processes (Diaz and Cabido 2001; Petchey et al. 2004; Petchey and Gaston 2006; Flynn et al. 2011). It is important to note that this approach identifies the potential for rare species to influence ecosystem functioning and does not directly measure ecosystem functioning in a realized community.

**Figure 1:**
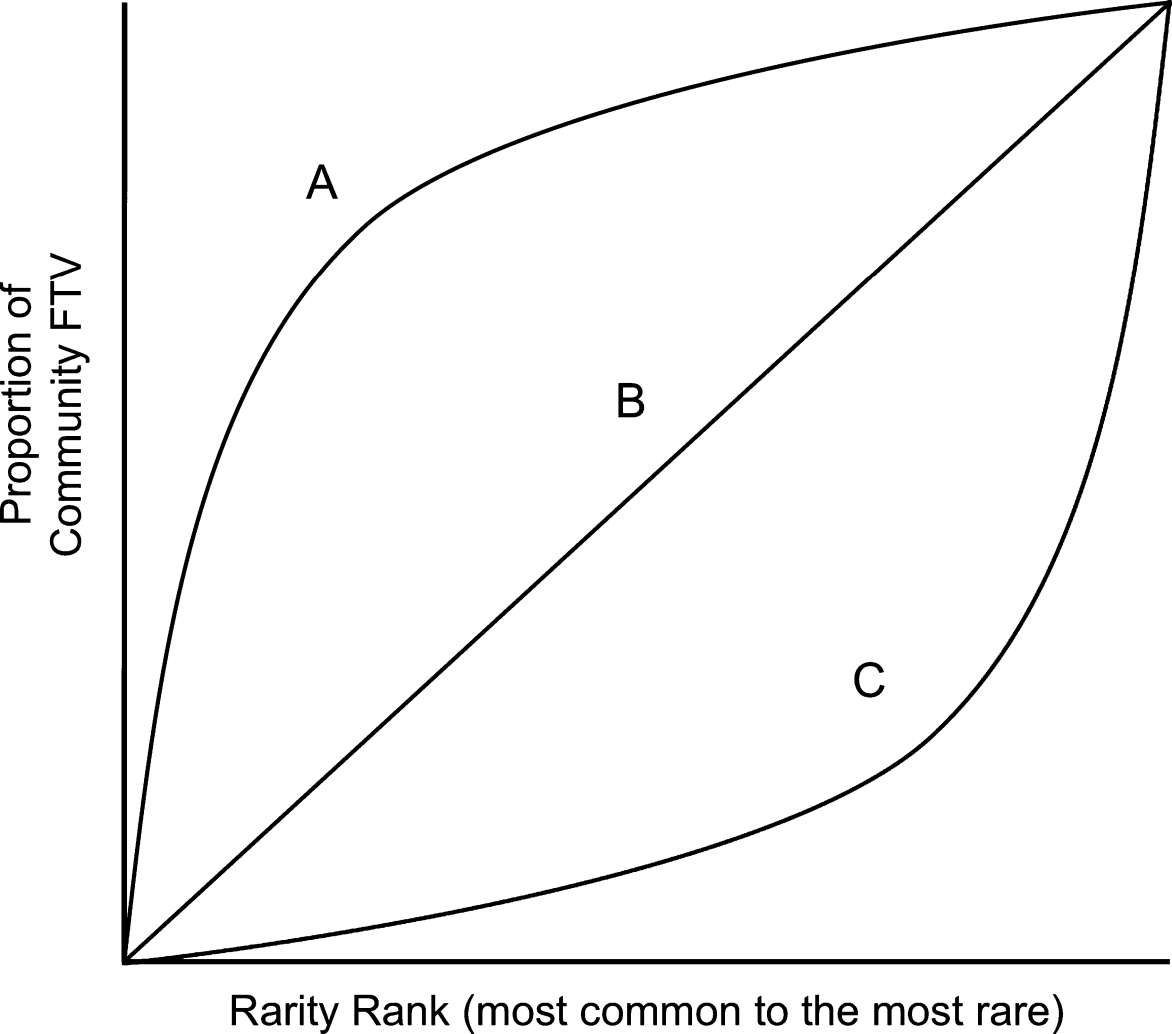
Three hypothetical ways in which rare species may contribute to community functional diversity. Species are ranked from the most common to the most rare on the *x*-axis. If rare species are redundant with those of common species, rare species will not contribute much to functional diversity (Curve A). If rare species and common species contribute to functional diversity to a similar degree, all species within the community contribute equally to functional diversity (Curve B). Finally, if rare species contribute more to functional diversity than common species, then much of a community's functional diversity can be explained by the rarest species (Curve C).

We discuss three ways in which rare species have been suggested to contribute to functional diversity (Naeem 1998; Tilman 1999; Loreau 2000). If the traits of rare species are redundant with those of common species (Fig. 1, Curve A), rare species may play a role in temporal complementarity by serving as substitutes for common species that suffer local extinction. This may occur if rare species perform similar functions as common species, but differ in their abilities to respond to environmental change and disturbance (Buckland et al. 1997; Walker et al. 1999; Diaz and Cabido 2001). If rare species contribute equally to functional diversity as common species (Fig. 1, Curve B) or if rare species contribute more to functional diversity than do common species (Fig. 1, Curve C), rare species possess functional traits that are unique to the community and may contribute to novel ecosystem functioning.

When discussing the influence of rare species, it is important to consider how rarity is defined because definitions vary across studies and conservation strategies. In BEF studies that consider dominance and rarity, mean abundance or frequency across communities is typically used (e.g., Lyons and Schwartz 2001; Smith and Knapp 2003; Hooper et al. 2005). However in conservation research, species are often defined as rare if they have small geographic ranges (e.g., Broennimann et al. 2005). We therefore used four widely used definitions of rarity (see Materials and Methods) to ensure broad applicability of our findings and to understand how different definitions of rarity may lead to varying results.

This study examines the potential influence of rare species on ecosystem functioning in grassland sites at Cedar Creek Natural History Area, MN. Specifically, we examine the contribution of rare and less common species to community functional diversity, when rarity is defined in one of four different ways. We argue that while this study does not specifically examine the effect that rare species have on a realized function, quantifying the influence of rare and less common species on community trait space is an important first step in understanding the impact that rare species may have on ecosystem function either presently or over time if their abundances increase due to environmental change.

## Materials and Methods

### Study site and organisms

**Figure 2:**
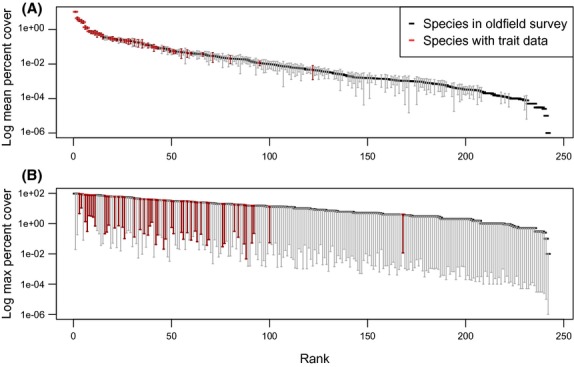
Rank-abundance plots of the 248 plant species present in the Cedar Creek oldfield survey, using two of the four definitions of rarity: (A) mean abundance for each species; error bars are ±1 SE and (B) maximum abundance for each species; bars show the range down to the mean abundance value. Species for which we have trait data, and are thus included in our analyses, are highlighted in red. Species are ranked from the most common to the most rare.

We examined the influence of rare and less common species' traits on the functional diversity of tall-grass prairie communities at Cedar Creek Ecosystem Science Reserve, MN. Abundance data for 248 plant species were collected from 1983 to 2002 every 5 years in a long-term observational study. In each year, hundred 1-m^2^ plots were randomly chosen in each of 19 abandoned agricultural fields (Knops and Tilman 2000). Of the 248 species in the survey data, sufficient trait data were collected for 46 species (trait data collection methods described below), which is approximately twenty percent of the species found in our study plots (Fig. 2). While data were available for some rare species, no trait data were available for the 123 most rare species, when rarity was defined by local abundance. Despite this, 28 of our species had mean abundances <10% of our ten most common species, and 18 of our species had mean abundances <5% of the ten most common species, demonstrating that our study species span the commonness-rarity range (Fig. S1). While our study does not explicitly consider the very rarest species in our analyses, we believe that identifying the relationship between relative rarity rank and functional diversity offers valuable insight into the potential role of the rarest species in ecosystem functioning. Such data limitations are inherent in most trait databases given the difficulty in collecting trait data for very rare species, which are difficult to locate, and we address the consequences of these limitations in the Discussion section.

### Quantifying rarity

Rarity results from several properties of species' distributions and has subsequently been defined differently across studies. Rabinowitz (1981) identified three key properties to rarity: (1) geographic range, (2) habitat specificity, and (3) local abundance. Our typology of rarity differs from that of Rabinowitz by focusing on the three axes of rarity, but not their interactions. Although some of our measures of rarity are correlated (see Table S1), we used each metric independently to facilitate comparison with studies that measure rarity based on only one metric.

#### Geographic range

The geographic range of each species was derived from the USDA PLANTS database (*n* = 43) and was defined as the number of US states and Canadian provinces in which the species is present. The smaller the geographic range of a species, the rarer it is assumed to be.

#### Habitat specificity

Habitat specificity was defined using coefficients of conservatism (CC), derived from the Floristic Quality Assessment Index (FQAI) of the State of Ohio (*n* = 41; Andreas et al. 2004); these values range from zero to ten, where zero represents species with wide-ranging ecological tolerances and ten indicates species with a high fidelity to a narrow range of habitats (Swink and Wilhelm 1979). Higher CC values represent rarer species.

#### Local abundance (mean and maximum abundance)

We quantified local abundance as the mean, nonzero abundance of each species across time within fields sampled at Cedar Creek (*n* = 46). Given that we are unsure whether zero abundances in our dataset were true (e.g., reflecting the genuine absence of a species in a census) or false (e.g., failed detection attributable to inadequate temporal or spatial sampling), we took the conservative approach of assuming zeros were false and thus excluded all zeros when quantifying mean abundances (Martin et al. 2005). We also examined the maximum abundance of each species within a plot across all years as the fourth rarity metric (*n* = 46). Lower mean and maximum abundances represented rarer species.

### Functional trait data

We selected four traits that are broadly reflective of the life-history strategies of plants and are key measures of plant physiology and primary productivity. These were (1) total plant mass, (2) root-to-shoot ratio, (3) leaf mass per area (LMA, g·m^−2^), and (4) leaf nitrogen (%N). Plant mass in grasslands largely determines canopy position and thus the light-capturing niche of a species, root-to-shoot ratio reflects relative investment in resource capture belowground, and LMA and %N form part of the “leaf economic spectrum”, where species exhibit a trade-off between high rates of photosynthesis and leaf tissue longevity (Garnier et al. 2001; Reich et al. 2003; Wright et al. 2004; Dahlgren et al. 2006). Trait data were collected at the Cedar Creek Ecosystem Science Reserve in the summers of 2004 and 2005, following methods as described in Cornelissen et al. (2003). For each species, at least six individuals were selected from oldfields, separated by a reasonable distance to ensure different genotypes were sampled. The selection of the four traits was made to represent unique contributions to grassland plant resource capture and growth, and to avoid high correlations between traits (such as several size-related traits or several tissue N concentration traits).

We assessed trait correlations with Pearson's correlation coefficients for each pair of our four traits. While some of our traits are significantly correlated (Fig. S2), each trait adds unique information to the functional diversity of the community and the total correlation is never >0.34 for any pair. We thus considered all four traits in our analyses. In addition, to examine whether our results were overly dependent on any one of our four traits, we conducted sensitivity analyses using each subset of three of the four traits. We found that our results were similar across all models, suggesting that our conclusions are fairly robust to the combination of traits used in our study (see Table S2). Finally, we assessed trait correlation with a principal component analysis, which showed that at least three (of the possible four) components were needed to describe 90% of the trait variation (Fig. S3). Analysis of the influence of species on convex hull volume across rarity measures (see below) was not significantly affected using ordination axes versus log-transformed trait values (Fig. S4).

### Analyses

To assess the effect of each species on community functional diversity, we calculated the community convex hull volume, namely the volume of the minimum convex polytope required to bound the n-dimensional trait values for all species (Cornwell et al. 2006). For a species to contribute to community convex hull volume, its trait values have to be greater than the outermost combination of trait values of the other species present in our community of 46 species. In other words, these trait values have to be the minimum or maximum value for the entire community's set of traits. We conducted two separate analyses: one where the influence of species on functional trait space was not abundance weighted and one where the influence was abundance weighted. While we present results from both analyses, we primarily focus on the non-abundance-weighted analysis because our study focuses on the potential of rare species to influence ecosystem function regardless of their current abundance (e.g., the abundance of rare species could increase over time due to environmental change). However, to examine the potential impact of rare species on current ecosystem functions that are influenced by abundance (e.g., biogeochemical functions), we include abundance-weighted convex hull analyses in the supplementary information using methods described in Clark et al. (2012).

To examine how contribution to trait space varied between rarer and more common species, we removed each species with replacement and calculated the subsequent absolute change in total convex hull volume from a hypothetical community that includes all 46 species for which we have trait data. Given that we do not have trait values available for all species in the community, calculating the contribution of each species to the entire species pool is the best way to estimate the average contribution of each species to all the communities where it is found. This convex hull volume is defined here as the functional trait volume (FTV) of the community and is used as a measure of functional diversity.

To identify whether rarer species contributed to FTV, species were ranked from the most common to the most rare (see Table S3) and a linear regression was performed on the contributions to FTV by rarity rank. While it is possible that the relationship between FTV and rarity rank is not exactly linear, we were only interested in whether rare species generally contribute more or less to FTV than more common species, which would broadly be captured by a linear regression. To determine whether the analysis was significant, we bootstrapped species' contribution to FTV and compared the observed slope from the original linear regression to the distribution of slopes produced by the bootstrapped analysis. An analysis was considered significant if the observed slope fell within the lowest or highest 2.5% of slope values produced by the bootstrap analysis. The analyses were performed for each of the four measures of rarity evaluated in this study, resulting in four separate analyses.

To understand whether each species' contribution to FTV was influenced by the specific communities in which it was found, we constructed 1111 random communities where both the species composition and the number of species found within a community were randomly varied. Species composition was uniformly distributed between 2 and 46 species and presence or absence was randomly assigned to each species. If the observed contribution of each species to FTV from the previous analysis fell within the extreme 2.5% of values generated from the null analysis, this indicated that species' contribution to FTV was influenced by the specific communities in which it was found. All analyses were conducted using R Statistical Software (version 2.9.1, http://www.r-project.org).

## Results

**Figure 3:**
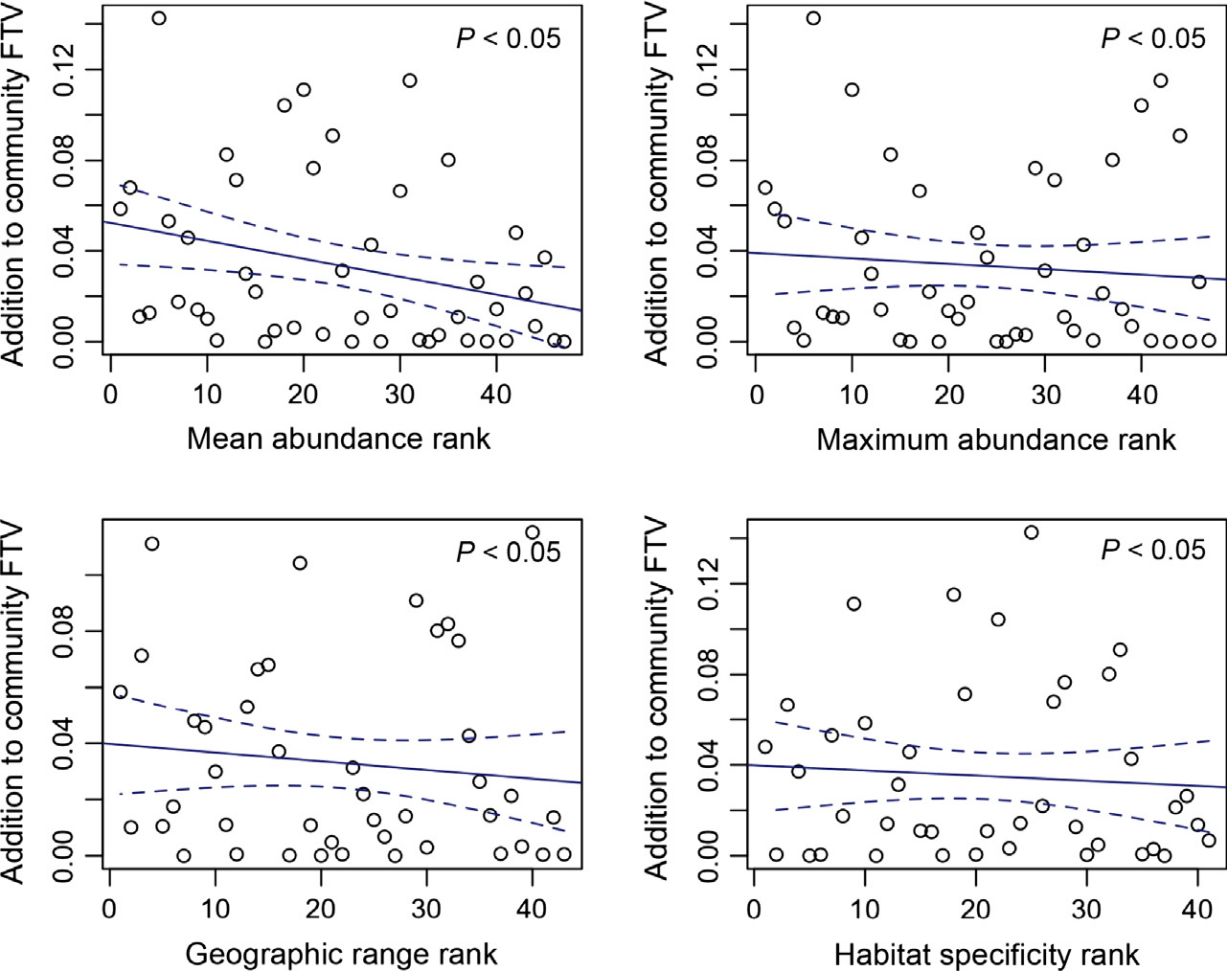
Contribution of each species to community functional trait volume (FTV) based on the four measures of rarity used in this study. Community FTV is the FTV of the 46 species considered in our study. Each point represents the mean absolute contribution of each species to the total community FTV. Species are ranked from the most common to the most rare along the *x*-axis. A regression line and its 90% confidence intervals are plotted for each graph. Each of the four plots show a negative trend between rarity and contribution to FTV; however, this trend is only significant when rarity is defined as mean abundance (*P* < 0.05).

Our non-abundance-weighted results indicate that the influence of rare species on FTV depends on how rarity is defined. Rare species influence FTV equally as common species when rarity is defined by maximum abundance, geographic range, and habitat specificity (Table 1, Fig. 3). These results correspond to our second hypothesis (Fig. 1, Curve B), where rare species contribute as much as common species to community functional diversity. However, in the case where rarity is defined by mean abundance, the rarer a species is, the less likely it is to contribute to community FTV (Table 1; *P* < 0.05, Fig. 3). This result corresponds to our first hypothesis (Fig. 1, Curve A), where rare species contribute similar trait values as common species and add little to community FTV.

**Table 1:** Values of regression slopes of rarity metrics as a predictor of contribution to community trait volume, as well as the *P*-values generated from bootstrap analysis for the four rarity metrics considered in this study. Significant values are starred

Rarity metric	Slope of regression	Bootstrap *P*-value
Mean abundance	−0.00086*	0.023*
Maximum abundance	−0.00027	0.269
Geographic range	−0.00031	0.217
Habitat specificity	−0.00023	0.335

Not surprisingly, our abundance-weighted regression analyses (see Fig. S5) show that rare species contribute significantly less to community FTV than common species (*P* < 0.05, Fig. S5) when rarity is defined by mean abundance, maximum abundance, and habitat specificity. These results suggest that rare species, even if they are functionally unique, may contribute little to community ecosystem function given their current observed low abundances. It is interesting to note that rare species contribute as much as common species to community FTV when defined by geographic range (Fig. S5).

Considering our null community analysis, the influence of rare species on FTV was insensitive to the species composition of the communities in which they were found. The observed contribution to FTV in natural communities for each of the 46 species for which we had trait data were not significantly different from contributions derived from null communities (Fig. S6).

## Discussion

We examined the relationship between relative rarity and contributions to trait space to identify the role that rarer species play in community functional diversity and possible ecosystem functioning. We find that the influence of rarer species depends on two factors. First, the way in which we define rare species changes the impact that rarer species have on functional diversity. Second, we find that when we weight contributions to trait space by abundance, rarer species contribute significantly less than more common species to community functional diversity. While these results cannot be directly linked to ecosystem function, they suggest that rare species may play a larger role in ecosystem functioning if their abundances increase due to environmental change. Yet, whether rare species offer novel functioning or are redundant depends on the way in which rarity is defined.

Rarer species have unique trait values and contribute to community functional diversity (Fig. 1, Curve B) when defined by maximum abundance, geographic range, or habitat specificity (Fig. 3). An increase in community trait diversity has been theoretically and empirically linked to increased ecosystem functioning (Petchey and Gaston 2006; Flynn et al. 2011; Reich et al. 2012). This may occur due to the selection effect, where greater diversity increases the chance that key traits important for ecosystem functioning are present in a given community, or due to niche complementarity, where a greater range of functional traits in a community results in the more efficient use of resources over space and/or time (Diaz and Cabido 2001). Several empirical studies have shown that less common species with unique traits have significantly contributed to ecosystem function. For example, plant communities that have lost less common species are more vulnerable to invasion compared with more species-rich communities (Lyons and Schwartz 2001; Zavaleta and Hulvey 2004). This is likely because communities with a greater number of species more effectively use resources, making it difficult for new species to become invasive. It is important to note that in these studies, rarer species played an important role in ecosystem functioning despite their low abundances.

When defined using mean abundance, rarer species contribute significantly less to functional diversity than do more common species (Fig. 1, Curve A). In this case, rarer species are functionally redundant, but have the potential to sustain future ecosystem functioning if they compensate for common species that go locally extinct due to environmental change (Walker et al. 1999; McLaren and Turkington 2011; MacDougall et al. 2013). Rarity defined by mean abundance was the only instance in which rarer species contributed significantly less to community functional diversity than more common species. Considering community assembly theory, this similarity in trait combinations between rarer and more common species may be explained by habitat filtering, where only species with particular trait combinations are able to survive in a given environment (Diaz et al. 1998; Cornwell et al. 2006; Kraft et al. 2008). Habitat filtering, as evidenced by the functional redundancy of rarer species, may only occur when defining rare species using local factors (e.g., mean local abundance) because the spatial scale of filtering may occur at localized scales (i.e., Cedar Creek grasslands) as opposed to regionally.

It is plausible that in many cases, rarer species may not influence current ecosystem functioning due to their low abundances (Grime 1998; Smith and Knapp 2003; Gaston 2011). This is suggested by our supplementary analyses using abundance-weighted measures of functional diversity (Fig. S5). We argue that even in these cases, it is still important to understand the effect that rarer species have on community trait space given that they may increase in abundance due to environmental change and influence future ecosystem functioning. For example, rarer species that are functionally redundant may contribute to community stability if they become more abundant and compensate for common species that are lost due to environmental change; MacDougall et al. (2013)show that in pyrogenic grasslands, diverse communities that include a large number of less common species are more resilient to interannual climate fluctuations, more resilient to the re-introduction of fire, and more resistant to invasion. Thus, even if rarer species do not significantly impact ecosystem function currently due to their low abundances, it is important to understand their contribution to community trait diversity because they may increase in abundance due to environmental change and influence future ecosystem functioning (Tilman and Downing 1994).

Although plant traits provide a useful means of assessing the potential for rare species to influence ecosystem functioning, the paucity of trait data for the rarest species limited our study. At Cedar Creek, a well-studied LTER site, trait data were available for only a fraction of the rare species (Fig. 2), irrespective of how one defined rarity. Furthermore, with trait data for only 46 species, our findings are likely to change whether we obtained trait data for all 248 species found in our study. In light of these limitations, we note that the 46 species for which we had trait data covered almost half the range from the most common to the most rare, allowing us to at least qualitatively explore the potential for rare species to influence ecosystem functioning. By including more species in our analyses, our key finding that the influence of rare species depends on how rarity is defined may not change. However, more specific findings, such as rare and common species contribute equally when rarity is defined by maximum abundance, geographic range, and habitat specificity, may change if trait values of extremely rare species differ significantly from the less to moderately rare species used in our study. Thus, we would like to highlight the methodological contributions of this study to understanding the role of rare species in ecosystem function as opposed to the ecological interpretations of our specific results.

In addition, trait selection certainly is an important consideration for all trait-based assessments of community structure. The selection of these particular traits was focused on traits important for contribution of plant species to grassland net primary productivity, but a different focus and thus different trait selection would require a separate analysis. For example, focusing the ecosystem function of nutrient cycling would require a different set of traits, such as fine root turnover and leaf nutrient resorption proficiency. The contributions of low-abundance, geographically sparse, or highly habitat specific rare plants could potentially be larger in such a case.

In conclusion, our analyses indicate that rarer species, whether defined by geographic range, maximum abundance, or habitat specificity, make important contributions to community trait space, suggesting that they have the potential to influence ecosystem functioning. When defined by mean abundance, rarer species may be valuable as replacements if they undergo compensatory growth for common species that go locally extinct, similar to the insurance hypothesis (Chapin et al. 1996). These two contributions to ecosystem function, either making a novel contribution to functional diversity or providing redundancy, offer support for the value of rare species and for examining the often overlooked contribution rare species make to ecosystem function. While such a trait-based approach is insightful and circumvents many of the challenges of working with rare species experimentally, the caveat is that trait data for rare species are likely to be, not surprisingly, rare. While we were unable to include the most rare species in our analyses, we have explored the impact of species that are much more rare than those commonly used in BEF experiments. Obtaining trait data for extremely rare species in the future will be valuable to assess the applicability of our findings to the full plant community at Cedar Creek. With these caveats in mind, our work supports the hypothesis that losing rarer species from a community could have profound impacts on community function, either presently or in the long term, if common species decline and rare species exhibit compensatory growth or are favored due to environmental change.

## Supporting Information

**Table S1.** Pearson correlations between each of the rarity measures used in our analyses.

**Table S2.** Sensitivity analysis examining all three-trait combinations from the four traits used in our analyses.

**Table S3.** Species names for all 46 species that were considered in our analyses.

**Figure S1.** Unscaled rank-abundance plots of the 248 plant species present in the Cedar Creek oldfield survey, showing mean abundance for each species; error bars are ±1 SE.

**Figure S2.** Pearson's correlation coefficients (r) between the four traits considered in our study: leaf nitrogen, leaf mass per area (LMA), root-to-shoot ratio, and plant mass.

**Figure S3.** Summary of principal component analysis of the four selected traits across the 46 species.

**Figure S4.** Principal component-based convex hull volumes, showing the influence of individual species on the change in hull volume.

**Figure S5.** Contribution of each species to abundance-weighted community functional trait volume based on the four measures of rarity used in this study.

**Figure S6.** Comparison of the contribution of each of the 46 species to functional trait volume compared with the contribution of the same species to 1111 constructed null communities.

## References

[b1] Andreas B, Mack J, McCormac J (2004). Floristic Quality Assessment Index (FQAI) for vascular plants and mosses for the State of Ohio.

[b2] Bracken MES, Low NHN (2012). Realistic losses of rare species disproportionately impact higher trophic levels. Ecol. Lett.

[b3] Broennimann O, Vittoz P, Moser D, Guisan A (2005). Rarity types among plant species with high conservation priority in Switzerland. Bot. Helv.

[b4] Buckland SM, Grime JP, Hodgson JG, Thompson K (1997). A comparison of plant responses to the extreme drought of 1995 in northern England. J. Ecol.

[b5] Cardinale BJ, Srivastava DS, Duffy JE, Wright JP, Downing AL, Sankaran M (2006). Effects of biodiversity on the functioning of trophic groups and ecosystems. Nature.

[b6] Chapin FS, Torn MS, Tateno M (1996). Principles of ecosystem sustainability. Am. Nat.

[b7] Clark CM, Flynn DFB, Butterfield BJ, Reich PB (2012). Testing the link between functional diversity and ecosystem functioning in a Minnesota grassland experiment. PLoS ONE.

[b8] Cornelissen JHC, Lavorel S, Garnier E, Diaz S, Buchmann N, Gurvich DE (2003). A handbook of protocols for standardised and easy measurement of plant functional traits worldwide. Aust. J. Bot.

[b9] Cornwell W, Schwilk D, Ackerly D (2006). A trait-based test for habitat filtering: convex hull volume. Ecology.

[b10] Dahlgren JP, Eriksson O, Bolmgren K, Strindell M, Ehrlen J (2006). Specific leaf area as a superior predictor of changes in field layer abundance during forest succession. J. Veg. Sci.

[b11] Diaz S, Cabido M (2001). Vive la difference: plant functional diversity matters to ecosystem processes. Trends Ecol. Evol.

[b12] Diaz S, Cabido M, Casanoves F (1998). Plant functional traits and environmental filters at a regional scale. J. Veg. Sci.

[b13] Duffy JE (2009). Why biodiversity is important to the functioning of real-world ecosystems. Front. Ecol. Environ.

[b14] Flynn DFB, Mirotchnick N, Jain M, Palmer MI, Naeem S (2011). Functional and phylogenetic diversity as predictors of biodiversity–ecosystem-function relationships. Ecology.

[b15] Garnier E, Laurent G, Bellmann A, Debain S, Berthelier P, Ducout B (2001). Consistency of species ranking based on functional leaf traits. New Phytol.

[b16] Garnier E, Cortez J, Billes G, Navas M, Roumet C, Debussche M (2004). Plant functional markers capture ecosystem properties during secondary succession. Ecology.

[b17] Gaston KJ (2010). Valuing common species. Science.

[b18] Gaston KJ (2011). Common ecology. Bioscience.

[b19] Griffin J, O'Gorman E, Emmerson M, Jenkins S, Klein AM, Naeem S, Bunker D, Hector A, Loreau M, Perrings C (2009). Biodiversity and the stability of ecosystem functioning. Biodiversity, ecosystem functioning, and human wellbeing: an ecological and economic perspective.

[b20] Grime J (1998). Benefits of plant diversity to ecosystems: immediate, filter and founder effects. J. Ecol.

[b21] Hillebrand H, Bennett DM, Cadotte MW (2008). Consequences of dominance: a review of evenness effects on local and regional ecosystem processes. Ecology.

[b22] Hooper D, Chapin F, Ewel J, Hector A, Inchausti P, Lavorel S (2005). Effects of biodiversity on ecosystem functioning: a consensus of current knowledge. Ecol. Monogr.

[b23] Hubbell S (1997). The unified neutral theory of biodiversity and biogeography: a synopsis of the theory and some challenges ahead.

[b24] Isbell FL, Losure DA, Yurkonis KA, Wilsey BJ (2008). Diversity-productivity relationships in two ecologically realistic rarity-extinction scenarios. Oikos.

[b25] Knops J, Tilman D (2000). Dynamics of soil nitrogen and carbon accumulation for 61 years after agricultural abandonment. Ecology.

[b26] Kraft NJB, Valencia R, Ackerly DD (2008). Functional traits and niche-based tree community assembly in an Amazonian forest. Science.

[b27] Lavorel S, Garnier E (2002). Predicting changes in community composition and ecosystem functioning from plant traits: revisiting the Holy Grail. Funct. Ecol.

[b28] Lewis O (2009). Biodiversity change and ecosystem function in tropical forests. Basic Appl. Ecol.

[b29] Loreau M (2000). Biodiversity and ecosystem functioning: recent theoretical advances. Oikos.

[b30] Lyons K, Schwartz M (2001). Rare species loss alters ecosystem function – invasion resistance. Ecol. Lett.

[b31] Lyons K, Brigham C, Traut B, Schwartz M (2005). Rare species and ecosystem functioning. Conserv. Biol.

[b32] MacArthur RH, Wilson EO (1967). The theory of island biogeography.

[b33] MacDougall AS, McCann KS, Gellner G, Turkington R (2013). Diversity loss with persistent human disturbance increases vulnerability to ecosystem collapse. Nature.

[b34] Martin TG, Wintle BA, Rhodes JR, Kuhnert PM, Field SA, Low-Choy SJ (2005). Zero tolerance ecology: improving ecological inference by modelling the source of zero observations. Ecol. Lett.

[b35] McLaren JR, Turkington R (2011). Biomass compensation and plant responses to 7 years of plant functional group removals. J. Veg. Sci.

[b36] Mouillot D, Bellwood DR, Baraloto C, Chave J, Galzin R, Harmelin-Vivien M (2013). Rare species support vulnerable functions in high-diversity ecosystems. PLoS Biol.

[b37] Naeem S (1998). Species redundancy and ecosystem reliability. Conserv. Biol.

[b38] Petchey OL, Gaston KJ (2006). Functional diversity: back to basics and looking forward. Ecol. Lett.

[b39] Petchey O, Hector A, Gaston K (2004). How do different measures of functional diversity perform?. Ecology.

[b40] Pimm S, Jones H, Diamon J (1988). On the risk of extinction. Am. Nat.

[b41] Pimm SL, Russell GJ, Gittleman JL, Brooks TM (1995). The future of biodiversity. Science.

[b42] Rabinowitz D, Synge J (1981). Seven forms of rarity. The biological aspects of rare plant conservation.

[b43] Reich P, Buschena C, Tjoelker M, Wrage K, Knops J, Tilman D (2003). Variation in growth rate and ecophysiology among 34 grassland and savanna species under contrasting N supply: a test of functional group differences. New Phytol.

[b44] Reich PB, Tilman D, Isbell F, Mueller K, Hobbie SE, Flynn DFB (2012). Impacts of biodiversity loss escalate through time as redundancy fades. Science.

[b45] Smith MD, Knapp AK (2003). Dominant species maintain ecosystem function with non-random species loss. Ecol. Lett.

[b46] Swink F, Wilhelm G (1979). Plants of the Chicago region.

[b47] Tilman D (1999). The ecological consequences of changes in biodiversity: a search for general principles. Ecology.

[b48] Tilman D, Downing JA (1994). Biodiversity and stability in grasslands. Nature.

[b49] Walker B, Kinzig A, Langridge J (1999). Plant attribute diversity, resilience, and ecosystem function: the nature and significance of dominant and minor species. Ecosystems.

[b50] Wilsey BJ, Polley W (2004). Realistically low species evenness does not alter grassland species-richness-productivity relationships. Ecology.

[b51] Wright IJ, Reich PB, Westoby M, Ackerly DD, Baruch Z, Bongers F (2004). The worldwide leaf economics spectrum. Nature.

[b52] Yachi S, Loreau M (1999). Biodiversity and ecosystem productivity in a fluctuating environment: the insurance hypothesis. Proc. Natl Acad. Sci. USA.

[b53] Zavaleta E, Hulvey K (2004). Realistic species losses disproportionately reduce grassland resistance to biological invaders. Science.

