# Supplementary figures and images for: The importance of rare species: a trait-based assessment of rare species contributions to functional diversity and possible ecosystem function in tall-grass prairies
Source: Ecol Evol. 2013 Dec 12;4(1):104–12. doi: 10.1002/ece3.915 (PMC3894892; doi:10.1002/ece3.915)

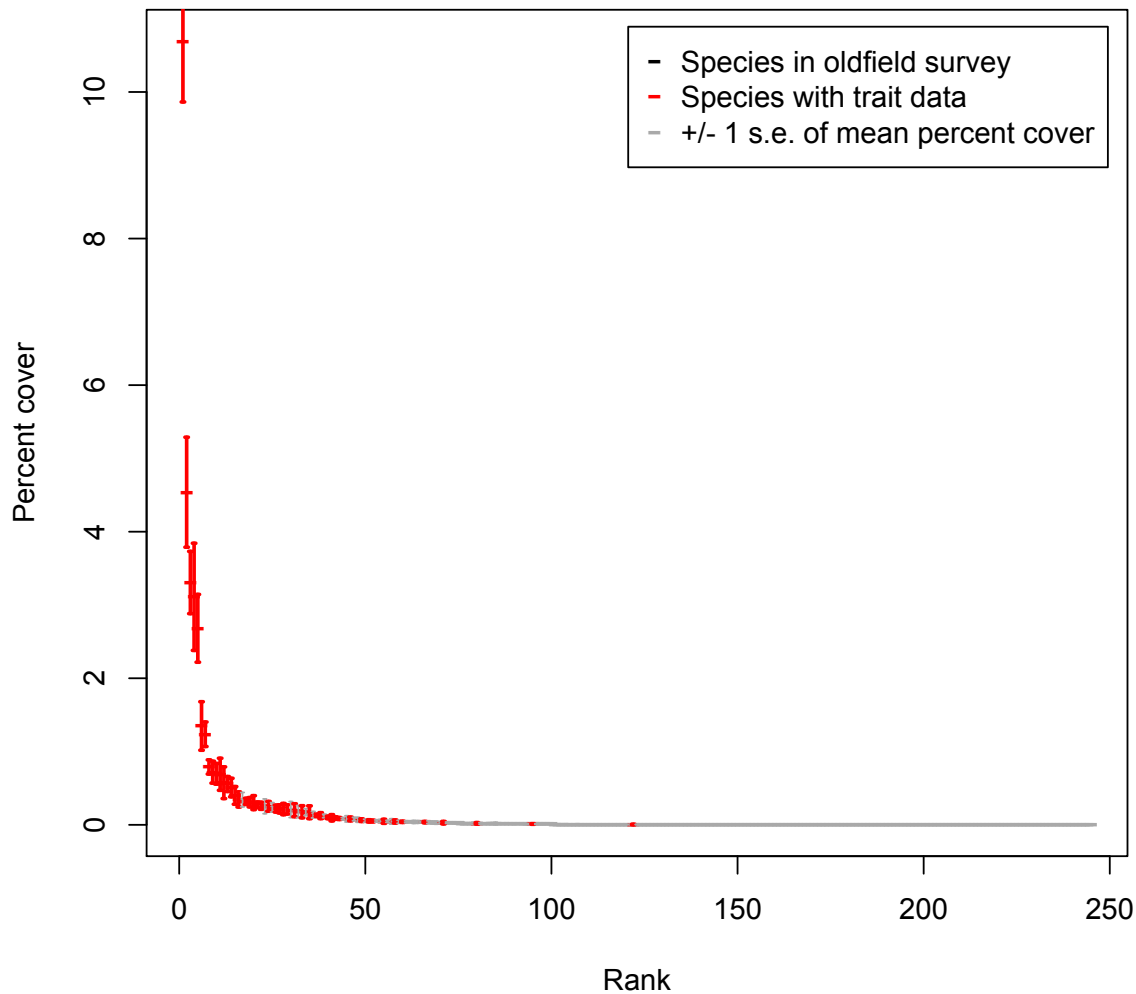

Supplement: Supplementary file 1 [file ece30004-0104-SD1.pdf]

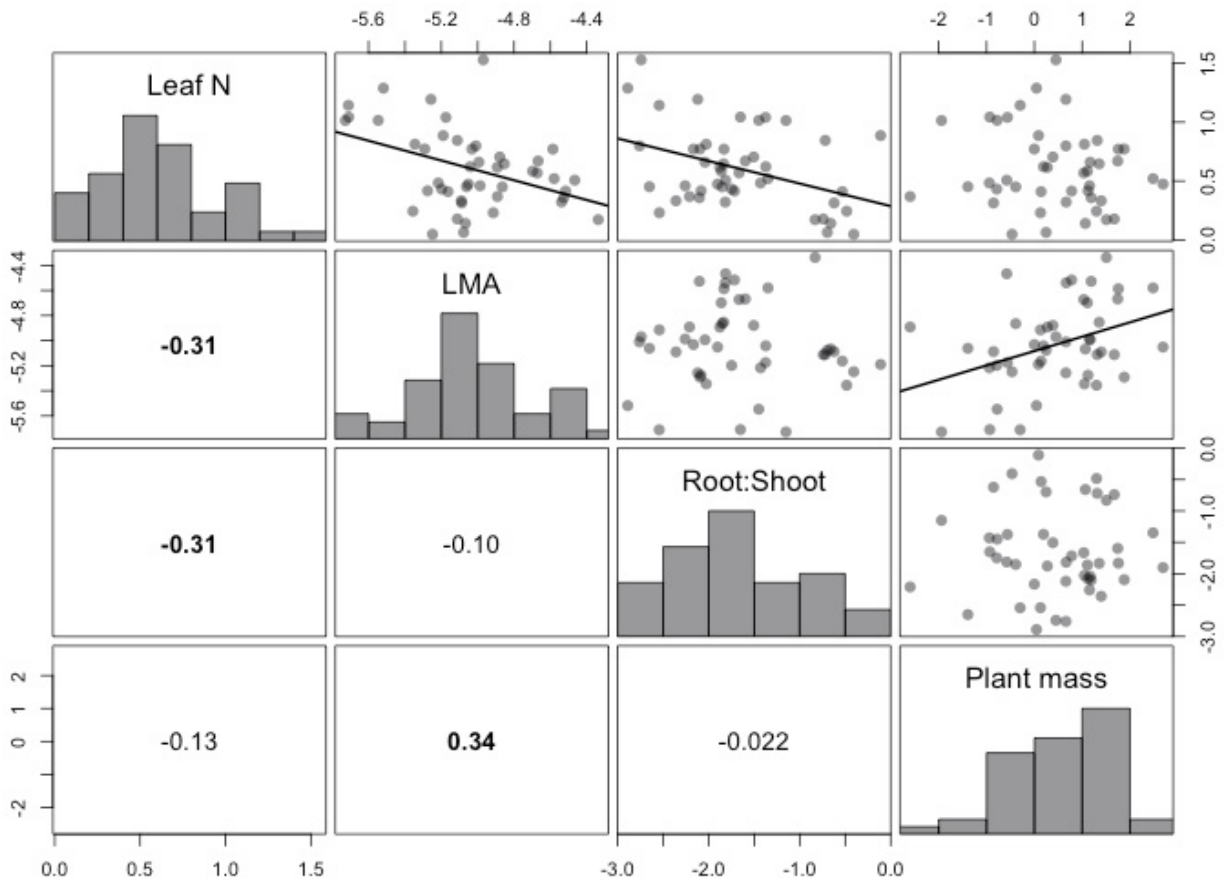

Supplement: Supplementary file 2 [file ece30004-0104-SD2.pdf]

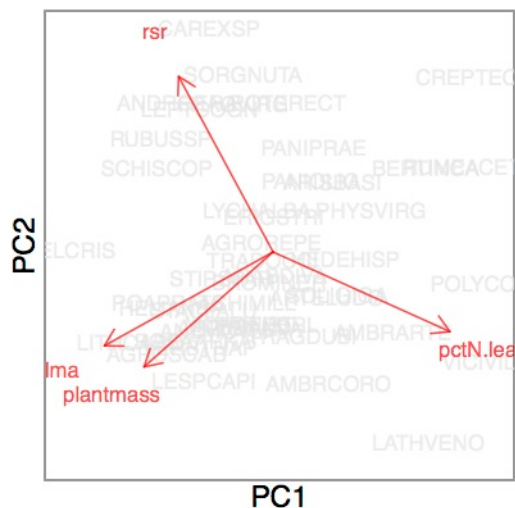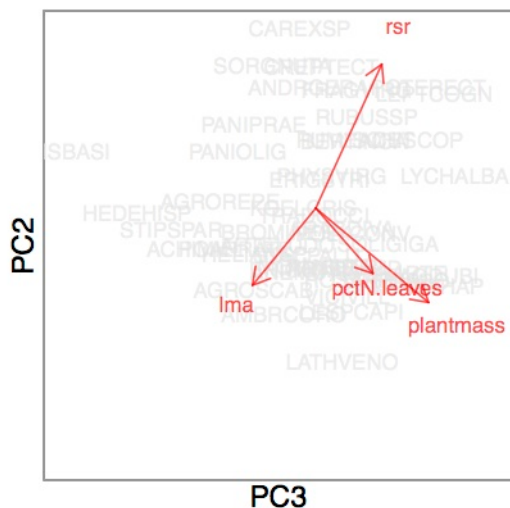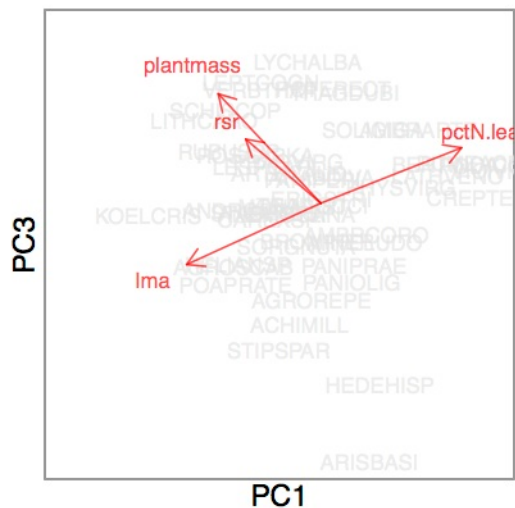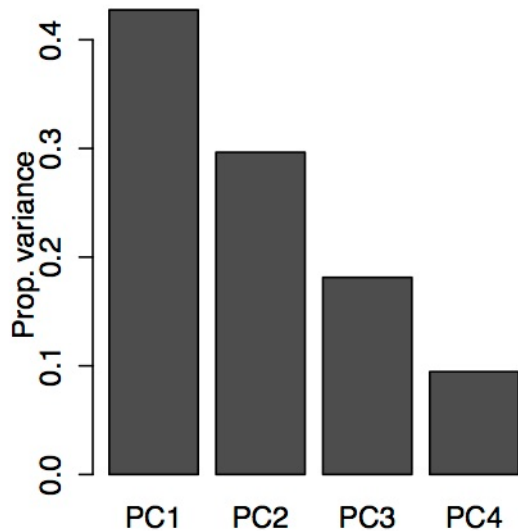

Supplement: Supplementary file 3 [file ece30004-0104-SD3.pdf]

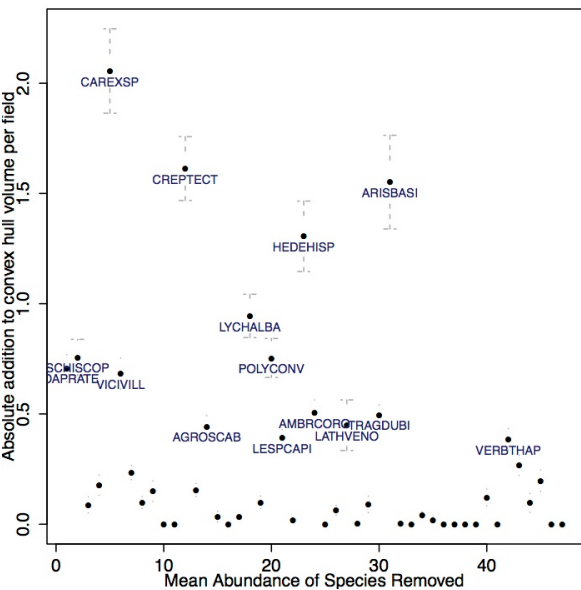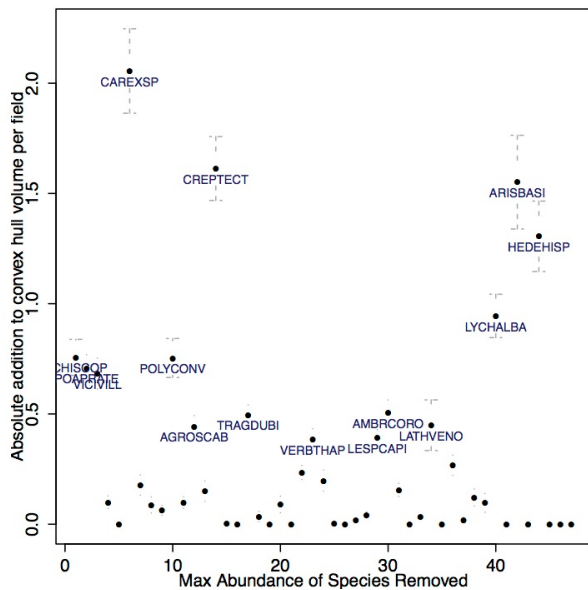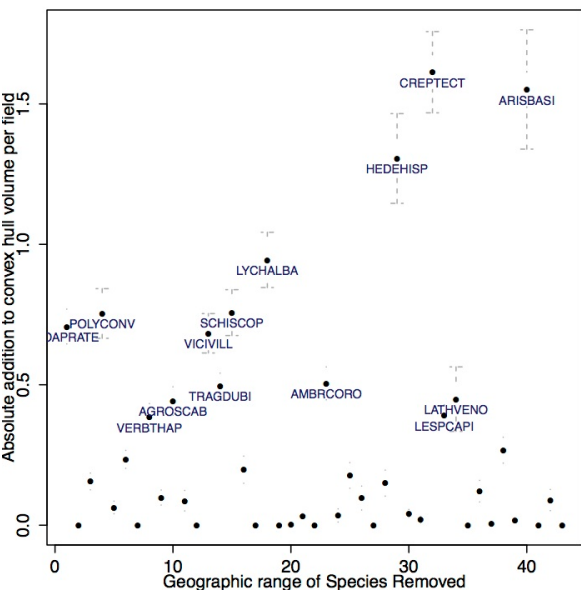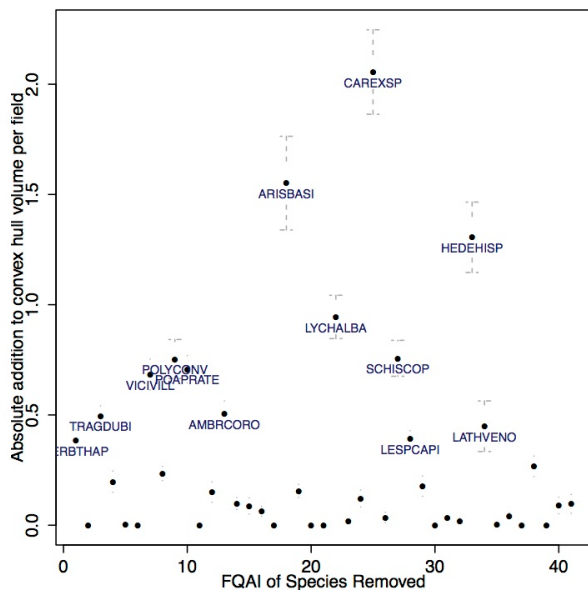

Supplement: Supplementary file 4 [file ece30004-0104-SD4.pdf]

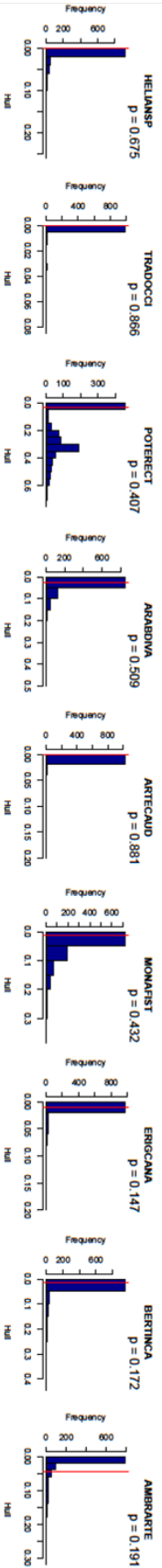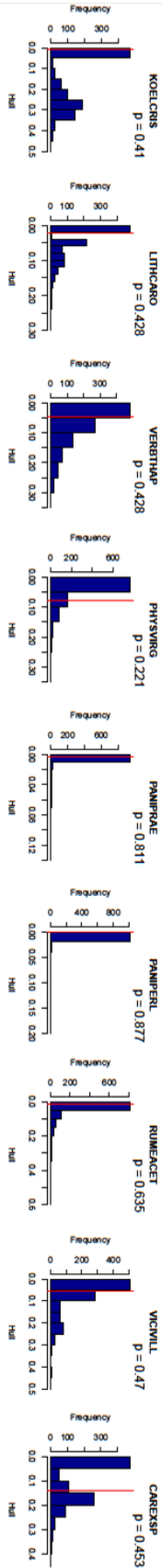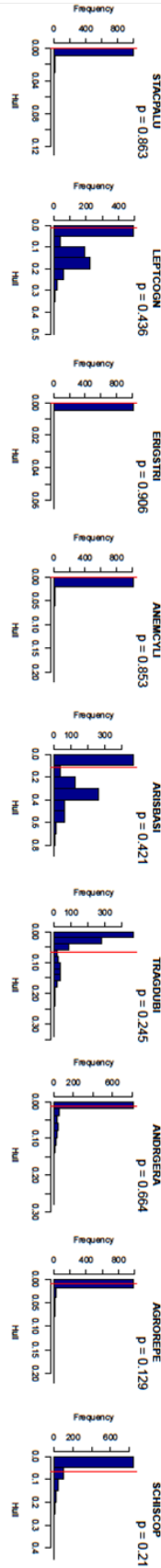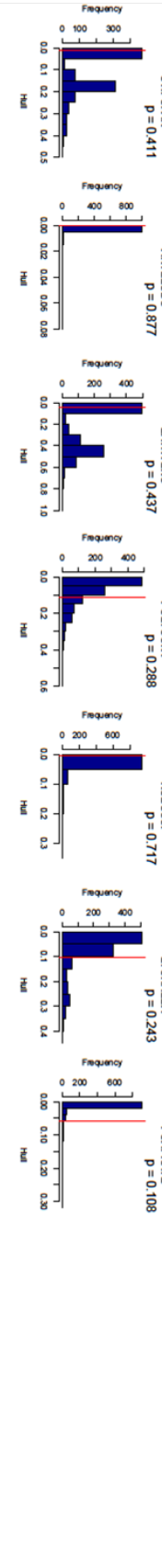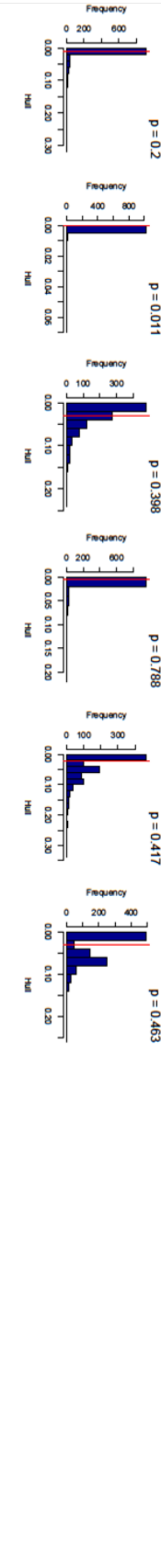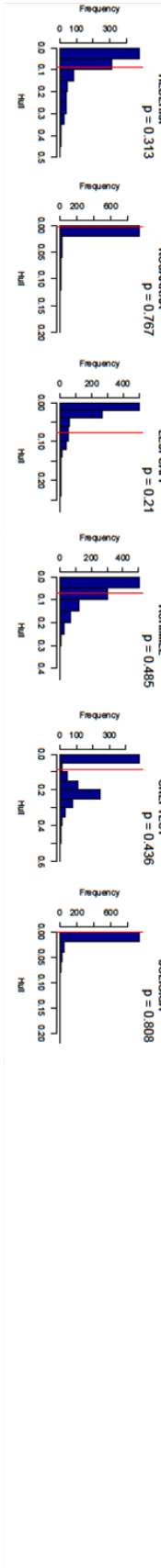

Supplement: Supplementary file 6 [file ece30004-0104-SD6.pdf]
