# Supplementary material for: The importance of rare species: a trait-based assessment of rare species contributions to functional diversity and possible ecosystem function in tall-grass prairies
Source: Ecol Evol. 2013 Dec 12;4(1):104–12. doi: 10.1002/ece3.915 (PMC3894892; doi:10.1002/ece3.915)
Supplement: Supplementary file 5 [file ece30004-0104-SD5.pdf]

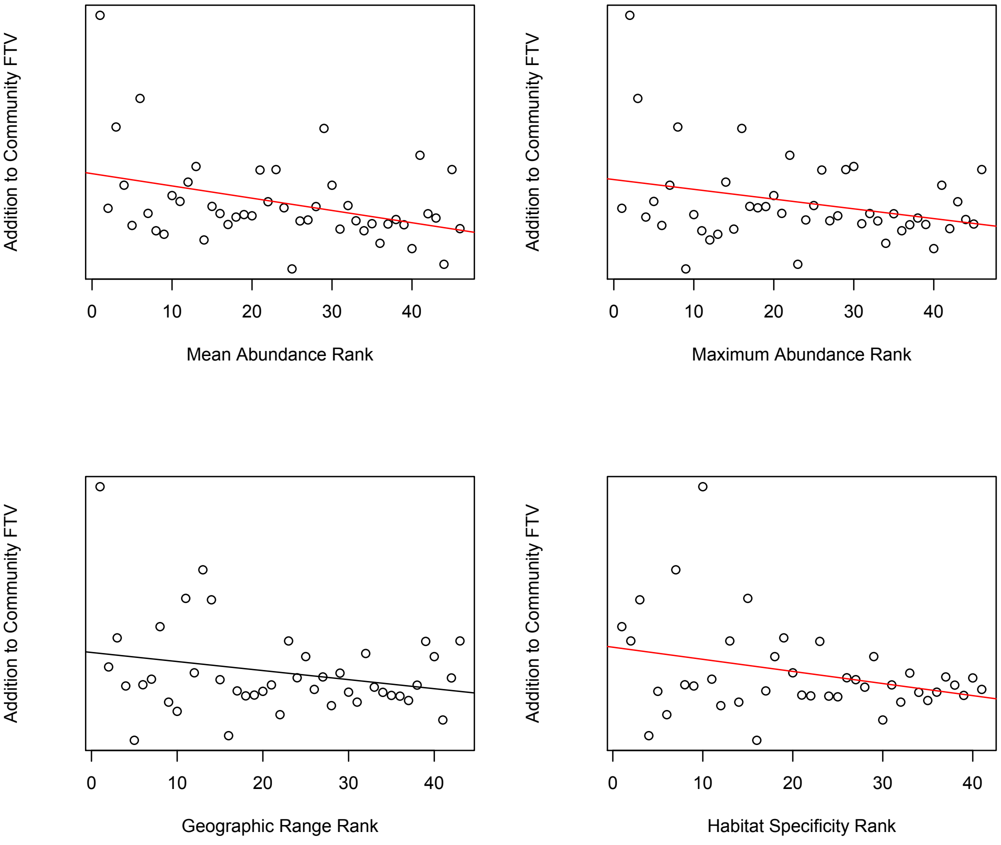

Addition to Community FTV
0
10
20
30
40
Mean Abundance Rank
Addition to Community FTV
0
10
20
30
40
Maximum Abundance Rank
Addition to Community FTV
0
10
20
30
40
Geographic Range Rank
Addition to Community FTV
0
10
20
30
40
Habitat Specificity Rank
