# Supplementary material for: The importance of rare species: a trait-based assessment of rare species contributions to functional diversity and possible ecosystem function in tall-grass prairies
Source: Ecol Evol. 2013 Dec 12;4(1):104–12. doi: 10.1002/ece3.915 (PMC3894892; doi:10.1002/ece3.915)

**Table S1.** Pearson correlations between each of the rarity measures used in our analyses. See text for details on rarity measures. Significant correlations (p < 0.05) are starred.

|  | Mean abundance | Maximum abundance | Geographic range | Habitat specificity |
| --- | --- | --- | --- | --- |
| Mean abundance | - | 0.70* | 0.50* | 0.29 |
| Maximum abundance |  | - | 0.58* | 0.31 |
| Geographic range |  |  | - | 0.63* |
| Habitat specificity |  |  |  | - |

**Table S2.** Sensitivity analysis examining all three-trait combinations from the four traits used in our analyses. The slope of the regression as well as its *p* value from the bootstrapped analysis are reported (significance at α = 0.05 marked with **, at α = 0.1 marked with *). Rare species significantly contribute less to FTV than more common species when rarity is defined by mean abundance (p < 0.10). There is some evidence that plant mass influenced our results since rare species contributed less to FTV when rarity was defined by maximum abundance and geographic range (p < 0.10), however, there was still higher significance when rarity was defined by mean abundance (p < .05).

| Trait removed from analysis | Rarity metric | Slope of regression | Bootstrap p value |
| --- | --- | --- | --- |
| Leaf mass | Mean abundance | -0.0024 | 0.025** |
|  | Maximum abundance | -0.0004 | 0.344 |
|  | Geographic range | -0.0005 | 0.343 |
|  | Habitat specificity | -0.0005 | 0.341 |
| Root to shoot ratio | Mean abundance | -0.0007 | 0.061* |
|  | Maximum abundance | -0.0006 | 0.100 |
|  | Geographic range | 0.0002 | 0.341 |
|  | Habitat specificity | -0.0002 | 0.346 |
| Plant mass | Mean abundance | -0.0006 | 0.008** |
|  | Maximum abundance | -0.0004 | 0.080* |
|  | Geographic range | -0.0004 | 0.061* |
|  | Habitat specificity | -0.0001 | 0.342 |
| Leaf nitrogen content | Mean abundance | -0.0015 | 0.049** |
|  | Maximum abundance | -0.0010 | 0.140 |
|  | Geographic range | -0.0005 | 0.331 |
|  | Habitat specificity | -0.0014 | 0.102 |

**Table S3.** Species names for all 46 species that were considered in our analyses. Rarity rank of each of the four measures of rarity for each of our species is listed, where 1 equals the most common, 46 equals the most rare, and NA identifies species-rarity rank combinations where data were unavailable.

| Species Name | Habitat Specificity Rank | Geographic Abundance Rank | Mean Abundance Rank | Max Abundance Rank |
| --- | --- | --- | --- | --- |
| *Achillea millefolium* | 19 | 3 | 13 | 30 |
| *Agrostis scabra* | NA | 10 | 14 | 13 |
| *Ambrosia artemisiifolia* | 15 | 9 | 8 | 12 |
| *Ambrosia psilostachya* | 15 | 23 | 23 | 29 |
| *Andropogon gerardii* | 29 | 25 | 4 | 7 |
| *Anemone cylindrica* | 35 | 37 | 31 | 16 |
| *Aristida basiramea* | 19 | 40 | 30 | 41 |
| *Artemisa campestris* | 15 | 22 | 36 | 34 |
| *Artemisia ludoviciana* | 15 | 20 | 27 | 24 |
| *Berteroa incana* | 15 | 28 | 9 | 14 |
| *Boechera grahamii* | 39 | 37 | 37 | 45 |
| *Bromus inermis* | 15 | 7 | 24 | 19 |
| *Carex sp.* | 25 | NA | 5 | 6 |
| *Crepis tectorum* | NA | 3 | 10 | 20 |
| *Digitaria cognata* | 25 | 37 | 39 | 37 |
| *Dichanthelium oligosanthes* | 31 | 21 | 16 | 32 |
| *Dichanthelium perlongum* | 39 | 28 | 32 | 25 |
| *Dichanthelium acuminatum var.*  *acuminatum* | 39 | 31 | 33 | 27 |
| *Elytrigia repens* | 15 | 11 | 3 | 8 |
| *Erigeron canadensis* | NA | 32 | 12 | 15 |
| *Erigeron strigosus* | 19 | 17 | 38 | 44 |
| *Fragaria virginiana* | 19 | 5 | 25 | 9 |
| *Hedeoma hispidum* | 33 | 29 | 22 | 43 |
| *Helianthus sp.* | NA | NA | 46 | 42 |
| *Hesperostipa spartea* | 41 | 42 | 28 | 19 |
| *Koeleria macrantha* | 41 | 28 | 43 | 38 |
| *Lathyrus venosus* | 35 | 34 | 26 | 33 |
| *Lespedeza capitata* | 29 | 33 | 20 | 28 |
| *Lithospermum caroliniense* | 39 | 38 | 42 | 35 |
| *Monarda fistulosa* | 22 | 20 | 35 | 31 |
| *Physalis virginiana* | 33 | 31 | 34 | 36 |
| *Poa pratensis* | 15 | 1 | 1 | 2 |
| *Polygonum convolvulus* | 15 | 5 | 19 | 10 |
| *Potentilla recta* | 15 | 16 | 44 | 23 |
| *Rosa arkansana* | 25 | 40 | 21 | 26 |
| *Rubus sp.* | NA | NA | 18 | 4 |
| *Rumex acetosella* | 15 | 6 | 7 | 21 |
| *Schizachyrium scoparium* | 29 | 16 | 2 | 1 |
| *Silene latifolia* | 22 | 20 | 17 | 39 |
| *Solidago gigantea* | 22 | 12 | 11 | 5 |
| *Sorghastrum nutans* | 29 | 25 | 15 | 18 |
| *Stachys palustris* | 31 | 41 | 40 | 40 |
| *Tragopogon dubius* | 15 | 43 | 45 | 46 |
| *Tradescantia occidentalis* | 15 | 14 | 29 | 17 |
| *Verbascum thapsus* | 15 | 9 | 41 | 22 |
| *Vicia villosa* | 15 | 14 | 6 | 3 |

**Figure S1.** Unscaled rank-abundance plots of the 248 plant species present in the Cedar Creek oldfield survey, showing mean abundance for each species; error bars are ± 1 s.e. Species for which we have trait data, and are thus included in our analyses, are highlighted in red. Species are ranked from the most common to the most rare. Our analyses included 28 species that had mean abundances less than 10% of the 10 most common species, and 18 species that had mean abundances less than 5% of the 10 most common species suggesting that we covered a wide range of rarity ranks in our study.

**Figure S2.** Pearson correlation coefficients (r) between the four traits considered in our study: leaf nitrogen, leaf mass per area (LMA), root to shoot ratio, and plant mass. Histograms of each trait are on the diagonal, scatterplots of the two-way pairings of traits are in the upper-right, and correlation values are in the bottom-left. Significant correlations are in bold.

**
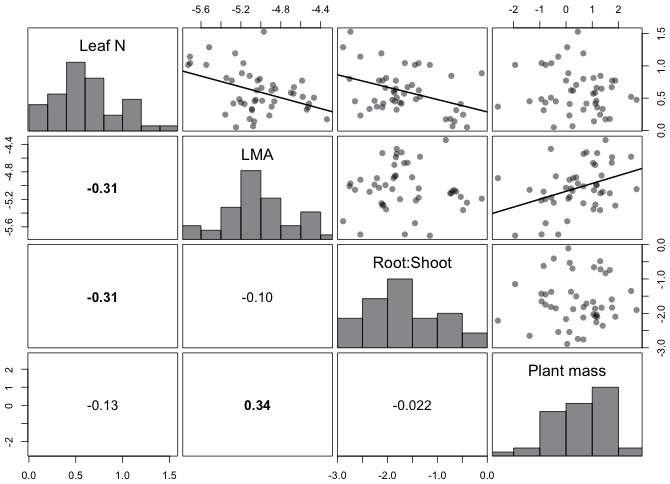
**

**Figure S3.** Summary of principal component analysis of the four selected traits across the 46 species. The first principal component (PC1) represents part of the leaf economics spectrum, with leaf mass per area (LMA) and leaf nitrogen concentration (pctN.leaves) negatively correlated with each other; this axis represented 47.7% of the variance (bottom right panel). Plant size (plantmass) was partially correlated with LMA for these grassland plants. PC2 separates plants along a root investment gradient, with graminoids at the high end; this axis represents and additional 26.9% of the variance. PC3 is driven mostly by plant size and captures 18.1% of the variance, for a total of 90.5% of the variation in these three orthogonal axes.

**
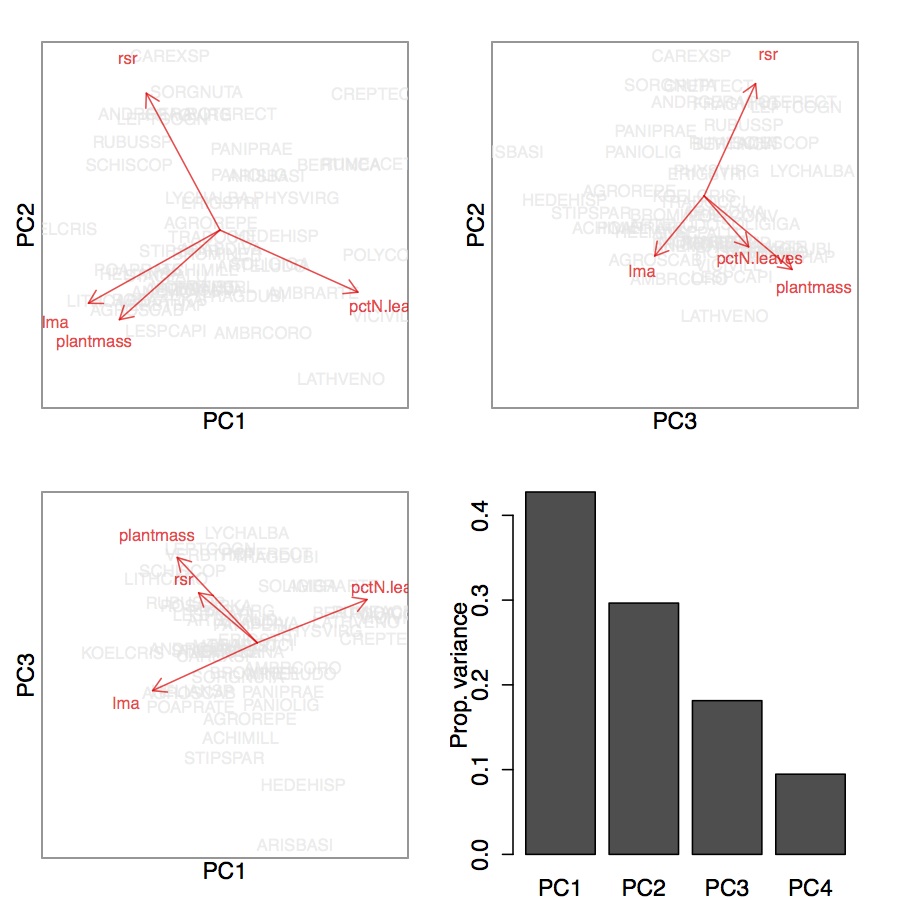
**

**Figure S4.** Principal component-based convex hull volumes, showing the influence of individual species on the change in hull volume. As for the non-reduced trait value convex hull volumes shown in Fig. 3, relationships between community trait space change and rarity are neutral or only weakly negative, reinforcing the result that rare species have important contributions to potential functional diversity.

**
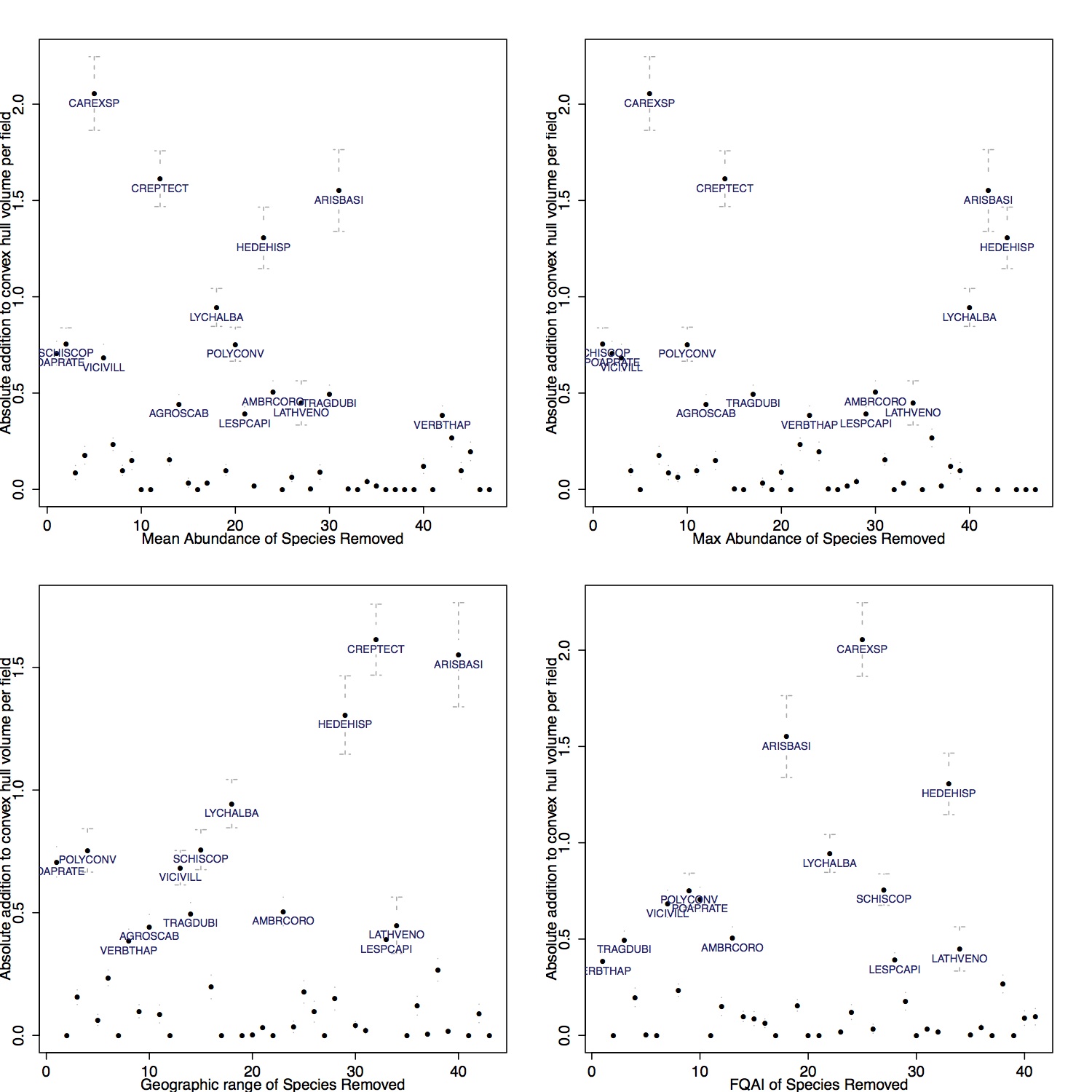
**

**Figure S5.** Contribution of each species to abundance-weighted community FTV based on the four measures of rarity used in this study. Community FTV is the functional trait volume of the 46 species considered in our study. Each point represents the mean absolute contribution of each species to the total community FTV. Species are ranked from the most common to the most rare along the x-axis. A regression line is plotted for each graph, and when significant, is highlighted in red. Regressions are significant when rarity is defined by mean abundance, maximum abundance, and habitat specificity (*p* < 0.05).

**Figure S6.** Comparison of the contribution of each of the 46 species to FTV compared to the contribution of the same species to 1,111 constructed null communities. Community FTV is the functional trait volume of the 46 species considered in our study. A significant *p* value indicates that the species is contributing more to its present community than is expected by chance and that our FTV analyses may be influenced by the particular composition of the communities in which the species is found. The results of this analysis suggest that no species is being influenced by the composition of the specific communities in which it is found given that all p ≥ 0.05.


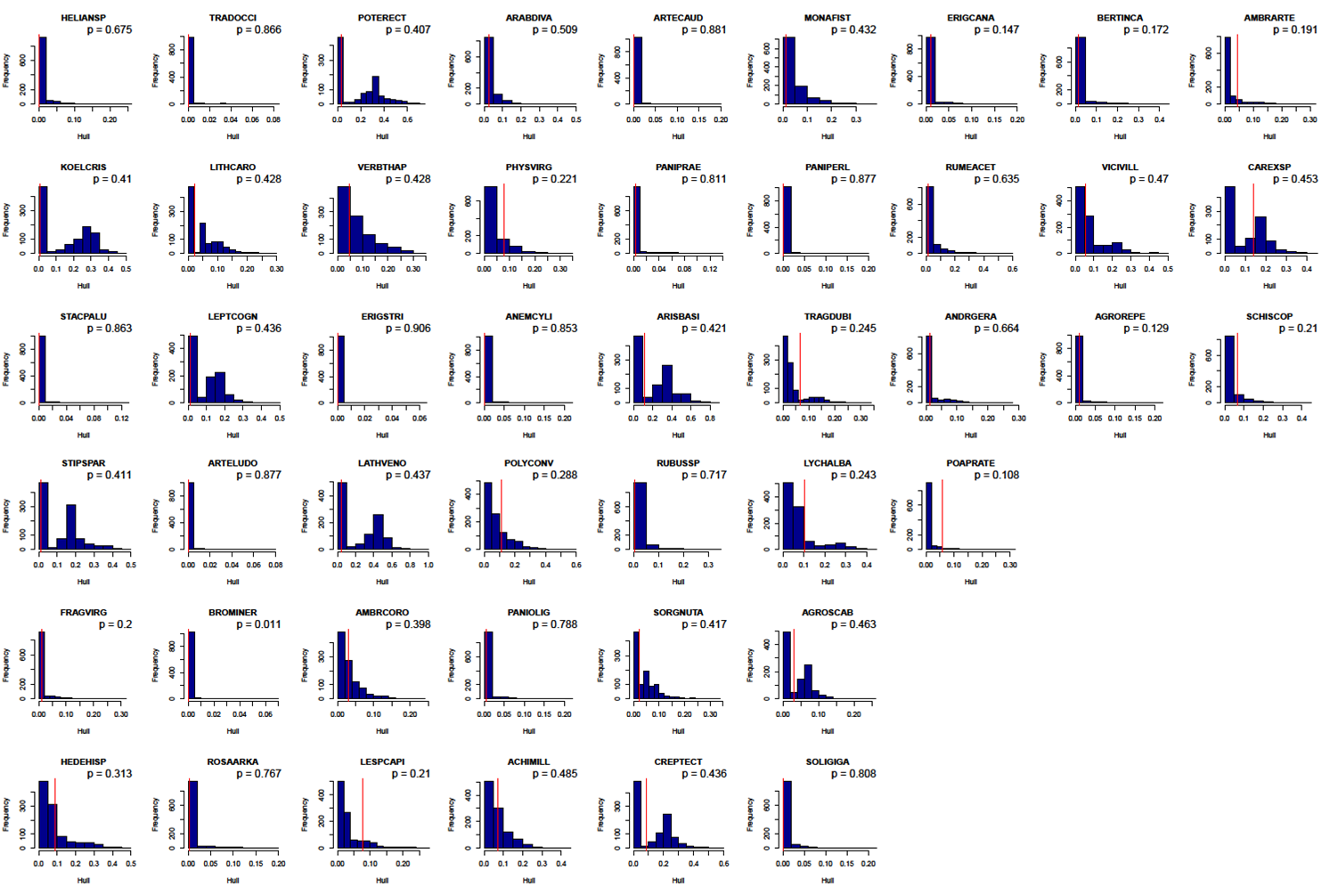

Supplement: Supplementary file 9 [file ece30004-0104-SD9.docx]
